# Molecular docking, network pharmacology and experimental verification to explore the mechanism of Wulongzhiyangwan in the treatment of pruritus

**DOI:** 10.1038/s41598-023-27593-5

**Published:** 2023-01-07

**Authors:** Lyu Anqi, Shan Shijun

**Affiliations:** 1 School of Medicine, Xiamen University, Xiamen, 361000 Fujian China; 2 Department of Dermatology, Xiang’an Hospital of Xiamen University, 2000 Xiang’an East Road, Xiamen, 361000 Fujian China

**Keywords:** Drug development, Preclinical research

## Abstract

Wulongzhiyangwan (WLZYW) is a Chinese prescription medicine for the treatment of pruritus, but its mechanism has not been clarified. The purpose of this study was to explore the mechanism of WLZYW in pruritus through network pharmacology analysis and experimental validation. The active components and corresponding targets of WLZYW were obtained from the Traditional Chinese Medicine Systematic Pharmacology (TCMSP) database. Pruritus-related targets were obtained from the GeneCards, TTD (Therapeutic Target Database), and DrugBank databases. The key compounds, core targets, main biological processes and signaling pathways related to WLZYW were identified by constructing and analyzing related networks. The binding affinity between WLZYW components and core targets was validated by AutoDock Vina software. In this study, RBL-2H3 cells were used to construct a degranulation model to simulate histamine-dependent pruritus. 10 chemical constituents, 235 targets and 3606 pruritus-related targets of WLZYW were obtained. Subsequently, 26 core targets were identified through analysis, VEGFA and AKT1 were the main candidates. A pathway enrichment analysis showed that overlapping targets were significantly enriched in the PI3K/AKT signaling pathway. A molecular docking analysis revealed tight binding of VEGF to three core compounds, kaempferol, luteolin and quercetin. Experiments showed that WZLYW inhibited mast cell degranulation, regulated VEGFa mRNA and protein expression levels by inhibiting PI3K/AKT and ERK1/2 signaling pathway activation. The mechanism of WZLYW in pruritus may be regulating VEGFa expression. Network pharmacology assays suggested that WLZYW downregulates VEGFa expression by regulating the PI3K/AKT and ERK1/2 signaling pathways in pruritis treatment.

## Introduction

Itch is a common sensory experience that is prevalent in patients with inflammatory skin diseases, as well as in those with systemic and neuropathic conditions. Itching that lasts 6 weeks or longer is considered chronic and its estimated lifetime prevalence is 22.6%^1^. It often leads to great personal stress and financial burden. The pathogenesis of pruritus is generally classified into histaminergic and nonhistaminergic pathways^2^. The former is mediated by histamine secreted primarily by mast cells, basophils and keratinocytes^3^. Mast cell activation causes histamine pruritus involves cell degranulation following the cross linking of immunoglobulin E (IgE) with high-affinity IgE receptors (FcεRI). Then, other influencing factors are released, such as histamine, prostaglandin, NGF, VEGFa, TNF-α and leukotriene^4^. When the patient is diagnosed with chronic pruritus of unknown etiology, treatment should be in principle according to T helper 2 (Th2) type inflammation^5^.

The Chinese Pharmacopoeia (2010 Edition) shows that Wulongzhiyangwan (WLZYW) is used to treat addictive rashes, rubella, and pruritus caused by the accumulation of heat rash on the skin, with symptoms that manifest as red spots, blistering rashes, and intolerable itching. WLZYW contains ten compounds, namely, Wushaoshe (*Zaocys dhumnades*), Fang feng (*Saposhnikovia divaricata*), Shechuangzi (*Cnidium monnieri* (L.) Cuss.), Kushen (*Sophora flavescens* Aiton)*,* Huangbai (*Phellodendron amurense* Rupr.), Cangshu (*Atractylodes lancea* (Thunb.) DC), Renshen (*Panax ginseng C. A. Meyer*), Mudanpi (tree peony bark), Shedanzhi (Snake bil), Bovis Calculus (*Bos taurus domesticus* Gmelin), and Danggui (*Angelica sinensis*). This formula is widely used in the treatment of pruritus in clinical practice.

Traditional Chinese medicine (TCM) have been used in China for thousands of years with good efficacy for certain diseases. Recently, researchers have gradually elucidated the effective components and targets of TCM. Network pharmacology is based on the similarity in structure and function between drugs, analyze disease-related genes in regulatory network of drug–component–target–disease, to explore the interactive relationships between target molecules and biological effector molecules in vivo. TCM are two or more mixed preparations, with complex regulatory networks. The ingredients in WLZYW formulation act on different targets and pathways, but some may act on the same target. In other words, the ingredients in the formula are synergistic. Network pharmacology integrates multidisciplinary technologies and contents, such as multidirectional pharmacology, computational biology, network analysis and related findings. The comprehensive network analysis of drug effects can be achieved by multitarget research strategies^6^. Furthermore, the research strategy used in network pharmacology is consistent with the research principles used to develop TCM^7^.

**Figure 1:**
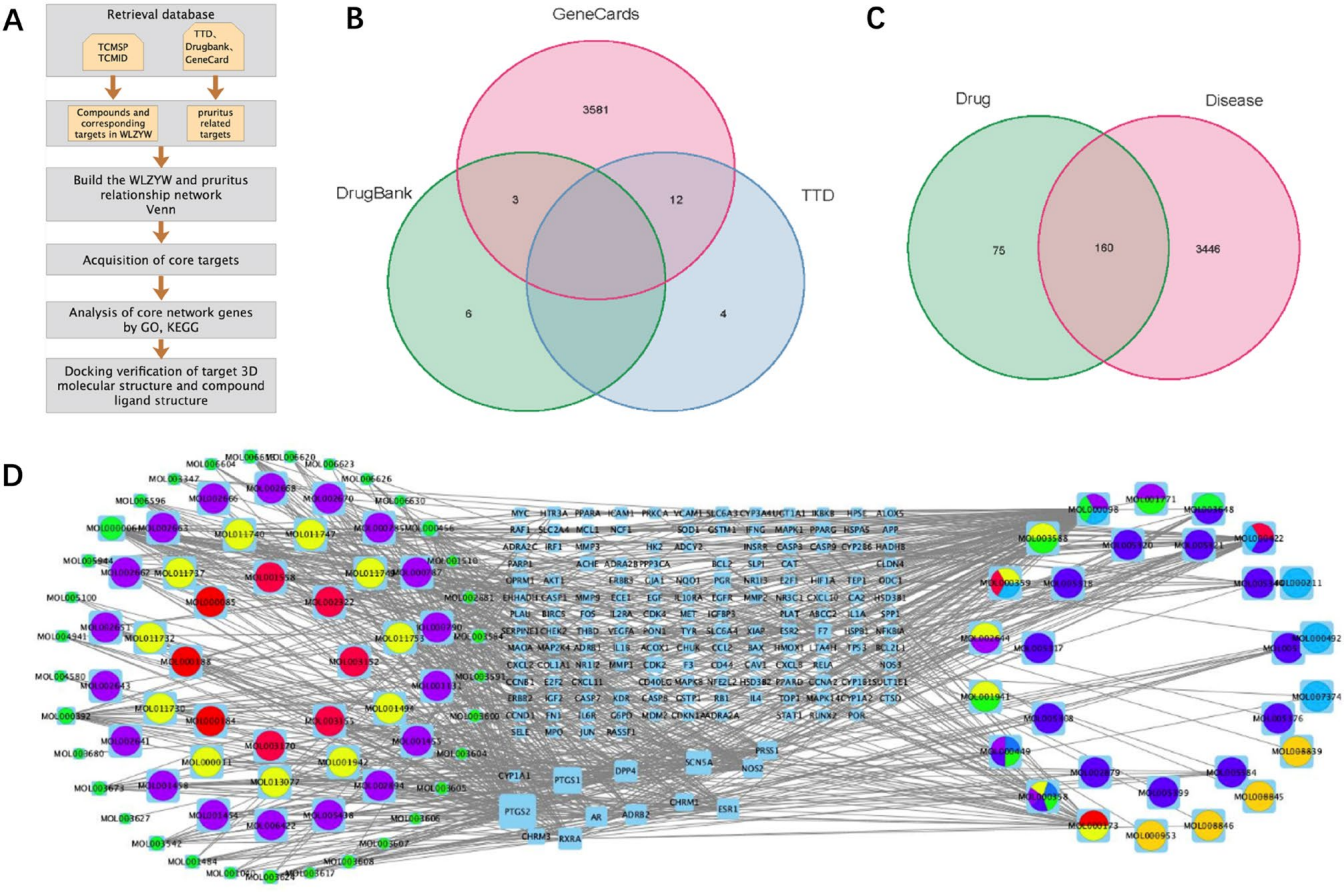
Regulatory networks of WLZYW components correspond to potential pruritus targets. (**A**) Schedule of Network Pharmacology. (**B**) Venn Diagram of Pruritus Targets. (**C**) The intersection of WLZYW components targets and pruritus targets. (**D**) WLZYW potential targets pool network. The light blue quadrilateral in the center represents 160 coincident targets of WLZYW and pruritus. The circles on each side represent the compounds, one color is one component. Visualization via Cytoscape 3.8.0.

In this study, we simulate the action network of WLZYW on the basis of manufacturing drug component target pathway network, selected the core drug components and the most likely drug targets, and validated the findings through molecular docking. The workflow diagram is shown in Fig. 1A. Then, we analyzed the mechanism of WLZYW in the treatment of pruritus and conducted in vitro tests to verify the conclusions.

## Results

### Regulatory network of potential targets corresponding to WLZYW components

Ten drugs in WLZYW formula correspond to 59 complex components, and 235 drug targets were selected by OB (Oral bioavailability) ≥ 30% and DL (Drug Likeness) ≥ 0.18 following the drug-like principle (Supplementary Table 1). 3660 pruritus-related targets were found by collating the targets obtained from TTD, Drugbank and GeneCard (Supplementary Table 2). Venn diagrams (Fig. 1B) were drawn by R. The intersection of therapeutic targets and pruritic related targets revealed 160 targets coincident, which were visualized and plotted by R and library Venn (Fig. 1C). Cytoscape was used to analyze the genes involved in WLZYW components and disease to construct regulatory network of compounds and targets (Fig. 1D). Evidently, one compound is able to regulate multiple targets, the same target maybe regulated by multiple compounds, indicating the presence of synergistic regulation of WLZYW components in the treatment of pruritus.

### Analysis of PPI interaction networks and core networks for coincident targets

**Figure 2:**
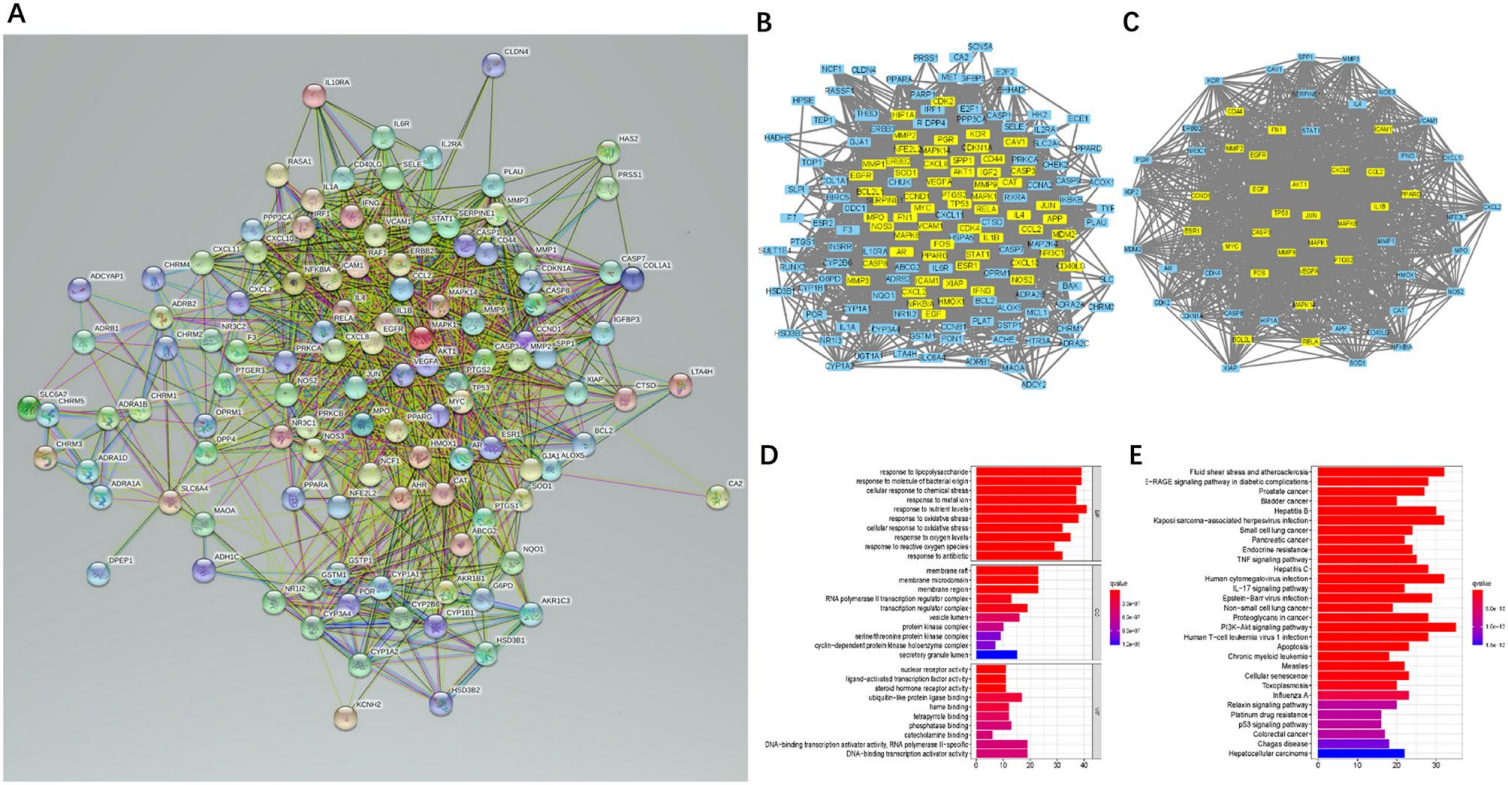
WLZYW core targets and pathway prediction. (**A**) PPI network of 160 potential targets. (**B**) Primary core network modules (yellow) identifying potential targets were analyzed via Cytoscape 3.8.0. (**C**) The core network module (yellow) was screened again from the core targets in (**A**). GO (**D**) and KEGG (**E**) maps of the core network. Drawing was performed by R 4.0.

To characterize the mechanism of WLZYW in the treatment of pruritus, a PPI network with 160 potential targets was constructed via cross acquisition. The hypergeometric test results of PPI were statistically significant (p < 0.05). The corresponding secondary protein interaction network was obtained (Fig. 2A), with 2955 edges and 160 nodes. CytoNCA plugin in Cytoscape 3.8.0 was used to analyze the targets in the PPI network. The core network obtained through this analysis represents some key features of PPI network and may have specific biological significance^8^. The network analysis of 160 targets showed an average centrality degree of 36.9375 for the compounds (Fig. 2A, Supplementary Table 3), and 60 genes greater than the mean were selected (Fig. 2B, Supplementary Table 4). With an average centrality degree of 41.73333, 60 genes were screened again, and 26 genes were greater than average (Supplementary Table 5). Then, core network of WLZYW compounds in the treatment of pruritus was constructed (Fig. 2C). The results indicated that WLZYW affects multiple targets in the treatment of pruritus. Notably, the network had 18 targets with centrality degrees greater than 50, and the most attractive were AKT1 (degree = 59) and VEGFa (degree = 59), both playing an important role in the network (Supplementary Table 5).

### GO and KEGG enrichment analysis of 26 core targets

**Figure 3:**
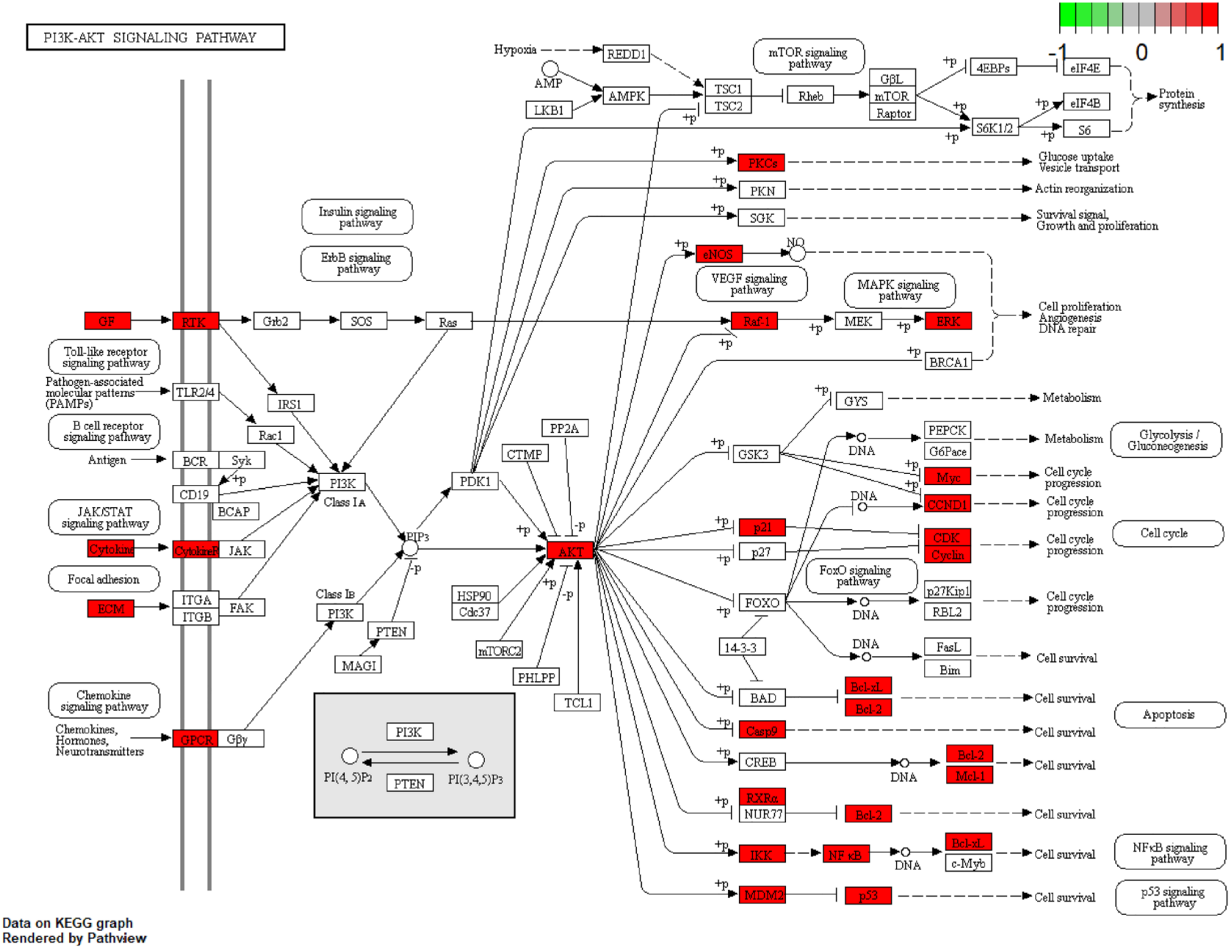
Pathway maps of potential targets for KEGG analysis. The red part represents the targets of WLZYW.

To illustrate the role, function, and signaling pathways of potential target genes, DAVID database visualization was used and comprehensive analysis was performed for gene annotations^9,10^. Gene Ontology (GO) and Kyoto Encyclopedia of Genes and Genomes (KEGG) analyses were performed for the 26 target genes in the PPI core network, resulting in 2489 GO enriched terms (Supplementary Table 6) and 163 KEGG pathways (Supplementary Table 7). After screening, the results were visualized by R 4.0 (Fig. 2D,E). The GO analysis showed that the mechanism of WLZYW in treating pruritus was mainly related to the biological process (BP), cellular component (CC), and molecular function (MF) categories: cellular response to oxidative stress caused by inflammation and regulation of gene expression by nuclear transcription factors. Moreover, KEGG enrichment analysis suggested that the mechanism of WLZYW in pruritus treatment was related to PI3K-Akt signaling pathway (hsa04151). In light of these data as well as the key targets combination, the PI3K-Akt signaling pathway was further visually analyzed in KEGG via Pathview (Fig. 3). Visual pathway map was used to show the mechanism of WLZYW in pruritus treatment, and the effects were primarily realized affecting cell proliferation and apoptotic progression. In terms of pruritus inflammation, the components in WLZYW had direct or indirect regulatory effects on inflammatory response.

### Validation of molecular docking

The PDB database archived the three-dimensional structure of biological macromolecules (proteins, nucleic acids, complex components AND sugars), which used by researchers to understand all aspects of biomedicine, from protein synthesis to the roles in health and disease. Among the 26 potential targets, AKTI and VEGFa showed the highest degree of centrality (degree = 59) and were closely related to the pathways indicated in KEGG analysis. Therefore, we searched the three-dimensional structure of AKT1 and VEGFa in PDB as the protein receptor in docking experiments. The interaction targets of WLZYW with AKT1 were identified by PPI network, and the corresponding compound ligands were luteolin, quercetin, and kaempferol. PubChem software was used to score and select the ligand structure with the smallest free energy. Docking of AKT1 and VEGFa receptor and their corresponding small-molecule drug ligands were performed by AutoDock Vina (opensource molecular docking program). Based on semiflexible docking, the interaction energy results are shown in Table 1.

**Table 1:** Interaction energy results obtained by AutoDock Vina.

Protein name	PDB ID	Compound	Binding affinity (kcal/mol)
VEGFA	6zfl	Kaempferol	− 6.8
VEGFA	6zfl	Luteolin	− 7.0
VEGFA	6zfl	Quercetin	− 6.9
AKT1	7nh5	Kaempferol	− 9.2
AKT1	7nh5	Luteolin	− 9.8
AKT1	7nh5	Quercetin	− 10.1

**Figure 4:**
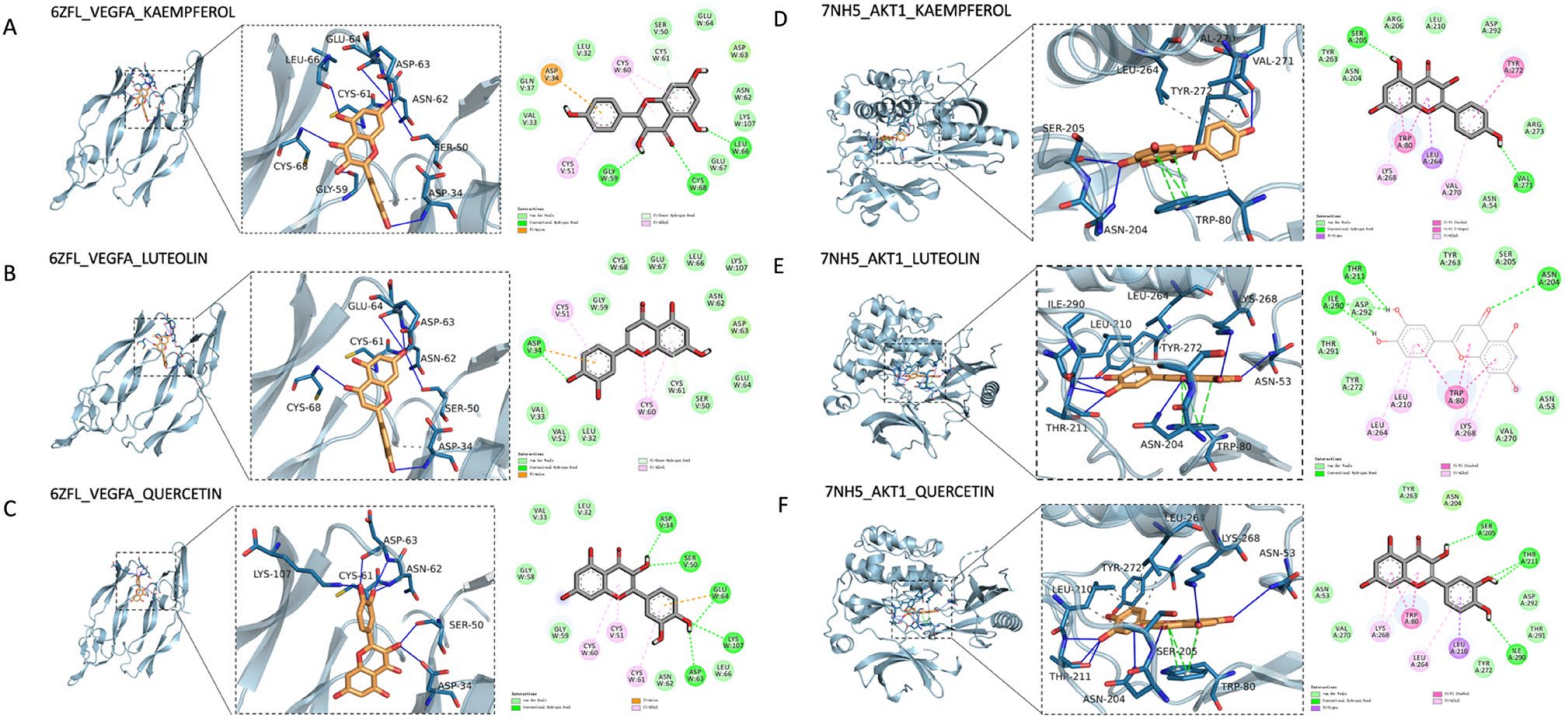
The compounds dock with VEGFA and AKT1. Molecular docking diagrams of VEGFA with kaempferol (**A**), luteolin (**B**), and quercetin (**C**). Molecular docking diagrams of AKT1 with kaempferol (**D**), luteolin (**E**), and quercetin (**F**).

Kaempferol, luteolin, and quercetin bound effectively to the active pockets of VEGFA protein. Their binding energies were − 6.8, − 7, and − 6.9 kcal/mol, respectively (Table 1). The results showed kaempferol formed hydrogen bonds with ASP34, SER50, GLY59, CYS61, ASN62, ASP63, GLU64, LEU66, and CYS68 of VEGFA protein ligand. We found that kaempferol also had hydrophobic interaction with ASP34 by analyzing three-dimensional interaction (Fig. 4A). Luteolin formed hydrogen bonds with ASP34, SER50, CYS61, ASN62, ASP63, GLU64, and CYS68 of VEGFA protein ligand and hydrophobic interaction with ASP34 (Fig. 4B). Quercetin interacted with VEGFA protein ligand at ASP34, SER50, CYS61, ASN62, ASP63, and LYS107 forming hydrogen bonds (Fig. 4C). Kaempferol, luteolin and quercetin interacted with the ligands, promoted their binding to active pockets of VEGFA, eventually formed complexes.

Kaempferol, luteolin and quercetin effectively bound to the active pockets of AKT1 protein with binding energies of − 9.2, − 9.8 and − 10.1 kcal/mol, respectively, all were lower than − 7.0 (Table 1). These data indicated that all three can spontaneously bind AKT1 protein pockets via hydrogen bonds, π–π parallel conjugation and hydrophobic interactions. Three-dimensional interactions analyses told us that kaempferol formed hydrogen bond interactions with ASN204, SER205 and VAL271/272 in ligands of AKT1 and hydrophobic interactions with TRP80, LEU264, VAL270 and TYR272 (Fig. 4D). Luteolin formed hydrogen bond interactions with ASN53, ASN204, SER205, THR211, LYS268 and ILE290 in AKT1 and hydrophobic interactions with TRP80, LEU210, LEU264 and TYR272 (Fig. 4E). Quercetin formed hydrogen bonds with ASN53, ASN204, SER205, THR211, LYS268 and ILE290 in ligands of AKT1 and hydrophobic interactions with LEU210, LEU264 and TYR272 (Fig. 4F). In addition, the benzene rings in kaempferol, luteolin, and quercetin formed π–π parallel conjugate with TRP80 aromatic ring of AKT1 protein. These interactions promoted ligands binding to active pockets of AKT1 protein, formed complexes.

### WLZYW inhibits DNP-IgE/BSA-induced degranulation of RBL-2H3 cells and downregulates VEGFa/TNF-α expression

**Figure 5:**
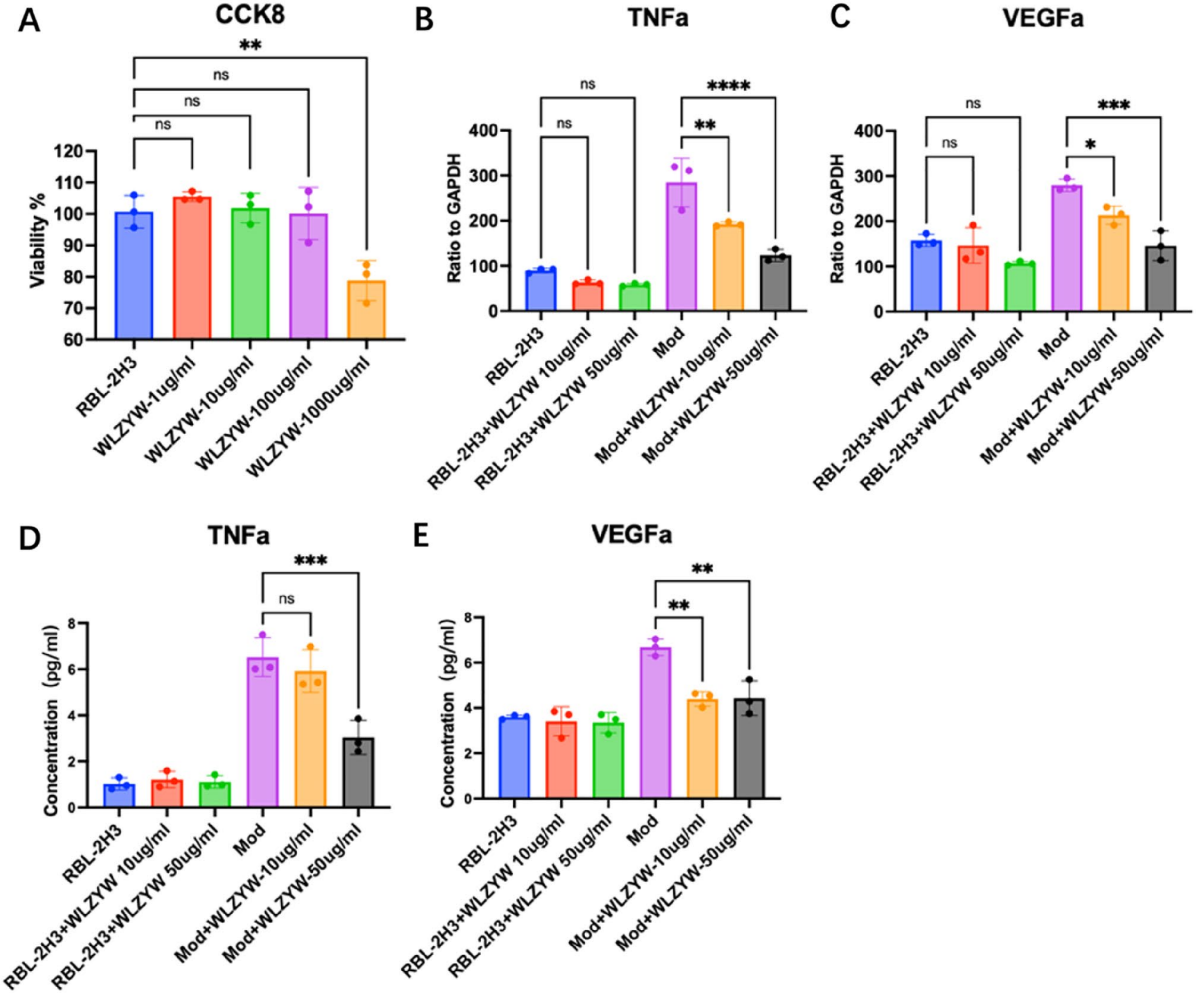
Effects of WLZYW on DNP-IgE/BSA-induced RBL-2H3 cells. CCK8 assay indicated that WLZYW was not cytotoxic to RBL-2H3 cells in certain range (**A**). QRT-PCR showed that WLZYW reduced mRNA expression of TNF-α (**B**) and VEGFa (**C**). Elisa showed that WLZYW decreased VEGFa protein expression (**E**), but only TNF-α protein expression decreased at high concentrations (**D**). Values represent mean ± SD from three independent experiments. *p < 0.05, **p < 0.01, ***p < 0.001, ****p < 0.0001, ns means no significant difference.

0.5 µg/mL DNP-IgE was used to induce RBL-2H3 cells for 24 h, and then, DNP-BSA (100 ng/mL) was added as the secondary stimulation to cause degranulation and release inflammatory mediators. WLZYW (1 µg/mL to 100 µg/mL) was not toxic to RBL-2H3 cells (Fig. 5A). For testing the inhibitory ability of WLZYW on mast cell activation, we used qRT-PCR to measure mRNA expressions of TNF-α and VEGFa in different groups. In the model group, the levels of TNF-α and VEGFa mRNA were higher, while in WLZYW groups (10 µg/mL and 50 µg/mL) the mRNA expressions of TNF-α and VEGFa were significantly decreased (Fig. 5B,C). However, the protein expression of TNF-α measured by ELISA did not exert significant inhibitory effect in 10 µg/mL group, but was significantly inhibited in 50 µg/mL group (Fig. 5D). Compared with the Mod group, VEGFa protein levels were significantly inhibited in both 10 µg/mL and 50 µg/mL groups (Fig. 5E).

### WLZYW downregulates VEGFa expression by inhibiting ERK1/2 and PI3K/AKT pathways

**Figure 6:**
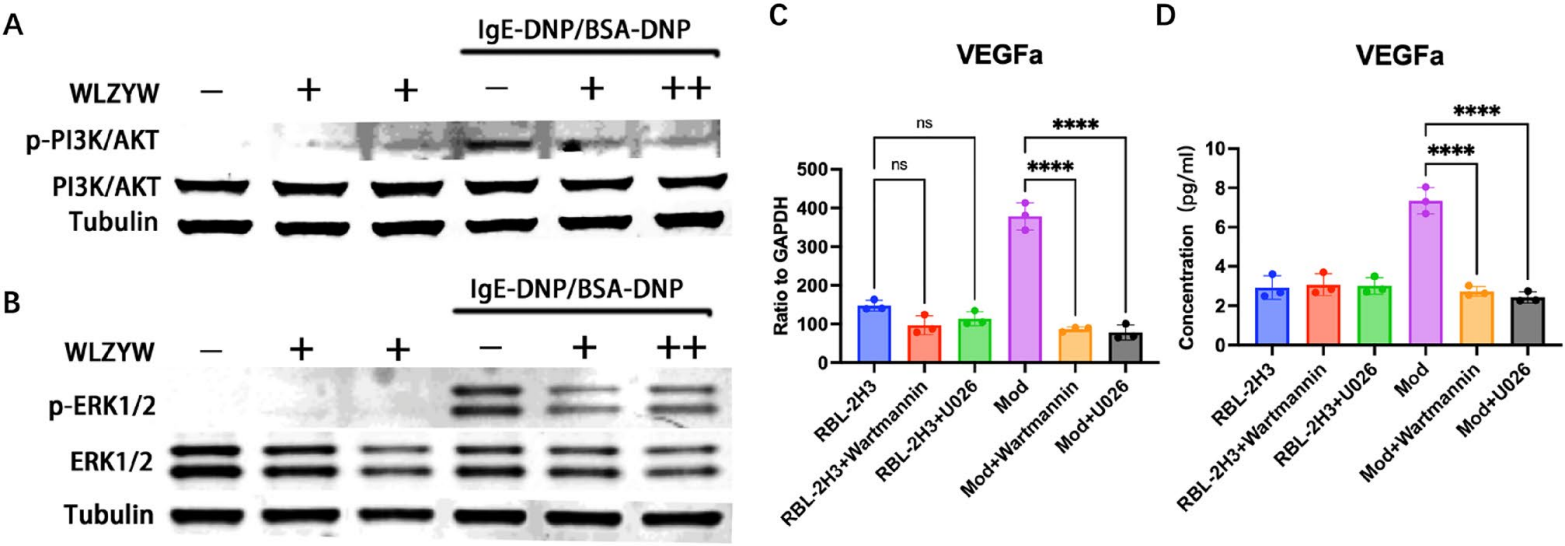
Effects of WLZYW on VEGFa and signaling pathways. WLZYW (50 μg/mL) inhibited the phosphorylation levels of p-PI3K/AKT (**A**) and p-ERK1/2 (**B**) in DNP-IgE/BSA induced degranulation model. After RBL-2H3 degranulation was inhibited by Wortmannin (1 μM) and U0126 (2 μM), VEGFa mRNA (**C**) and protein (**D**) levels were significantly downregulated. Values represent mean ± SD from three independent experiments, ****p < 0.0001, ns means no significant difference.

We performed WB to detect the expression levels of ERK1/2 and PI3K/AKT pathways associated with RBL-2H3 activation. Our results suggested that in the model group, the phosphorylation of PI3K/AKT and ERK1/2 pathways were higher, while in WLZYW treatment groups, they were significantly reduced (Fig. 6A,B). To examine the correlations between VEGFa and the two pathways, we used wortmannin (PI3K/AKT inhibitor) and U0126 (ERK/2 pathway inhibitor) to inhibit RBL-2H3 cell degranulation, and found that VEGFa mRNA and protein expressions were significantly reduced (Fig. 6C,D). The expression of VEGFa was dependent on the activation of PI3K/AKT and/or ERK1/2 pathway, and WLZYW could significantly inhibited the activation of them.

## Methods

### The compounds composition search of WLZYW

WLZYW contains ten herbs, namely, Wushaoshe-*Zaocys dhumnades*, Fangfeng-*Saposhnikovi ioconducta divaricata *(*Trucz.*), Shechuangzi-*Cnidium monnieri *(L.)* Cuss.,* Kushen-*Sophora flavescens Alt.,* Huangbai-*Phellodendron amurense Rupr*., Cangshu-*Atractylodes Lancea *(*Thunb.*)* DC*, Renshen-*Panax ginseng C. A. Meyer*, Mudanpi-*Tree Peony Bark*, Shedanzhi-*Snake bil*, Bovis Calculus-*Bos taurus domesticus Gmelin*, and Danggui-*Angelica sinensis*. They were searched in TCMSP database^11^ for drug screening, with OB ≥ 30% and OL ≥ 0.18, and obtained the targets name of all compounds. Symbol matching of target names were performed by human-derived gene information from Uniprot database for multi-database information docking.

### Search for pruritus-related targets

The retrieval of pruritus-related targets resulted in the collection of different genes associated with pruritus from the following resources. (1) Therapeutic Target Database (TTD)^12^: the keyword was pruritus, 16 pruritus-related targets were found. (2) GeneCards: the keyword was pruritus, 3596 pruritus-related targets were found. (3) DrugBank Online^13^: the keyword was pruritus, 9 pruritus-related targets were found.

### Acquisition of coincident targets

Pruritus targets and WLZYW targets were intersected by R 4.0.2 to generate coincident targets. Core target screening and analysis were performed by STRING database^14^. Finally, targets with score less than average were removed, after two times the core targets were selected.

### Protein–protein interaction data

Protein–protein interaction (PPI) data were obtained from Mutual Interaction Gene/Protein Search Tool. The coincident targets were entered into a database with species restricted to Homo sapiens, and confidence values were higher than 0.4. The hypergeometric test was performed via the phyper function of R 4.0.2.

### Network construction

Network construction was performed by two networks in this study. WLZYW compounds were connected with corresponding targets to establish compound-target network, network of pruritus targets was constructed by searching pruritus-related targets in different databases. The compound-targets network was crossed with the pruritus-related targets network to construct PPI network. Genes that did not cross were removed. A potential target–protein interaction network was constructed, and analyzed using modules to advance the core network. The networks were all made by Cytoscape 3.8.10, a software package used in the visualization and analysis for networks. For easily interpret large biological datasets, biological information is often visualized as graphs, such as nodes and edges. Nodes represent biomolecules, and edges between nodes represent certain relationship.

### GO and KEGG pathway analysis

DAVID (Database for annotation, visualization, and integrated discovery), functional enrichment analysis and KEGG (Kyoto Encyclopedia of Genes and Genomes) pathway enrichment analysis for the targets networks of pruritus genes corresponding to WLZYW were performed. In addition, Pathview was used to obtain visualized KEGG images^10,15^. The GO database includes BP, CC, and MF terms, were used to identify possible biological mechanisms based on high-throughput genomic or transcriptomic data^16^. KEGG is a reference knowledge base of biological interpretation for genome sequences and other high-throughput data. In addition, the bio-conductor cluster Profiler (org, Hs, Eg, db, DOSE), enrich plot, and pathview were used to analyze potential target modules for GO and KEGG pathway analysis. These five R packages were also applied in the enrichment analysis of gene clusters^17^.

### Molecular docking simulation

The SDF formats of compounds were downloaded from Pubchem data and imported into Chemdraw 3D. The energy was minimized using MM2 module to obtain the lowest energy, and saved as mol2 file. VEGFA and AKT protein structures were downloaded from the Protein Data Bank (PDB) database with PDB IDs of 6zfi and 7nh5, respectively. Subsequently, Pymol was used for visualization respectively. Mgtools 1.5.6 was used to dehydrate, hydrogenate, calculate charge and combine non-polar hydrogen, the ligands and receptors were respectively stored as PDBQT. Autodock vina 1.1.2 was used to dock ligand with receptor, and the parameters were shown below. The high rated were visualized by PYMOL and Discovery Studio.

### Cell culture and induce

RBL-2H3 cells were purchased from Procell (CHN). RBL-2H3 were cultured in DMEM medium containing 10% fetal bovine serum, 100 U mL^−1^ penicillin and 100 μg mL^−1^ streptomycin. The cells were cultured in a 37 °C, 5% CO_2_ incubator. RBL-2H3 (0.5 × 10^6^ cells/mL) grew in multi-well plates. After the cells fused to 75%, added DNP-IgE (MCE, USA) for 24 h, then washed with PBS. Cells and supernatants were collected after stimulation with 100 ng/mL DNP-BSA (Sigma, USA).

### CCK8 assay detect cell proliferation

CCK8 assay was performed to detect cell viability and proliferation. RBL-2H3 cells were seeded in the 96-well culture plates with 1 × 10^5^ cells/well and incubated for 24 h at 37 °C and 5% CO_2_. Cells were exposed to 1–1000 μg/mL WLZYW and further incubated for 24 h. The medium was replaced with 100 µL fresh medium containing 10% CCK8 (GLPBIO, CHN), and cells were incubated for 1.5 h. The OD (450 nm) absorbance value in each well was determined by spectrophotometer (Thermo, CHN). Cell viability rate (%) was calculated.

### Quantitative real-time PCR (qRT-PCR)

Cells were seeded in 6-well plate culture dish. When cells fused to 75%, added DNP-IgE (MCE, USA) for 24 h, washed with PBS, and replaced with WLZYW (10 μg/mL or 50 μg/mL). Cells were collected after stimulation with 100 ng/mL DNP-BSA for 10 min. Total RNA was extracted from cells using RNeasy Mini Kit (Qiagen Hilden, GER), and 1000 μg template RNA was transcribed into cDNA by Superscript III RT-PCR Kit (Vazyme, CHN). Then, the cDNA was qRT-PCR via SYBR GREEN (Bimake, USA). The primer sequences are: TNF-α, Forward-CATCCGTTCTCTACCCAGCC; Reverse-CATCCGTTCTCTACCCAGCC. VEGFa, Forward-ACGAAAGCGCAAGAAATCCC; Reverse-GCAACGCGAGTCTGTGTTTT. GAPDH, Forward-TGAGATCAACGTGTTCCAGTG; Reverse-ACCAGATGAAATGTGCCCC.

### Enzyme-linked immunosorbent assay (ELISA)

RBL-2H3 cells were sensitized with IgE (0.45 µg/mL) for 24 h and then treated with WLZYW (10 or 50 µg/mL) for 30 min. After cells were attacked by 100 ng/mL DNP-BSA for 4 h, supernatant was collected and the levels of TNF-α and VEGFa (Elabsciences, CHN) were determined with ELISA kit according to the instructions.

### Western blotting (WB)

The proteins extracted from cells were measured with BCA Protein Assay Kit (G-CLONE, Beijing, CHN). 25 μg protein samples were boiled at 90 °C for 7 min, then loaded and subjected to electrophoresis in 12% FuturePAGE (ACE, USA) gels at 160 V for 20 min. The membranes were probed with anti-PI3K/AKT antibody (CST, USA), anti-PI3K/AKT antibody (CST, USA), anti-p-ERK1/2 antibody (CST, USA), anti-ERK1/2 antibody (CST, USA), and mouse monoclonal antibody against tubulin (Abclonal CHN) overnight at 4 °C. IRDye 680RD Donkey anti-Mouse (LI-COR USA) and IRDye 800CW Donkey anti-Rabbit (LI-COR USA) was used as secondary antibody. The visualization of protein bands was accomplished by Li-cor Odyssey infrared imaging system (LI-COR USA).

### Statistical analysis

All experiments were repeated at least three times independently. Date was presented with mean ± SD, and statistical analysis was performed using Prism 8 (GraphPad, San Diego, CA). *t* test, one-way ANOVA and Dunnett’s multiple comparisons test were used. In all analyses, p values less than 0.05 were considered significant.

## Discussion

Pruritus is the most common complaint encountered by dermatologists, and it causes psychological stress and insomnia. Moreover, in patients with atopic dermatitis, it results in skin barrier damage. Pruritis aggravates the progression of skin disease and increases sensitivity to antigens^18^. Unfortunately, targeted treatments are limited, current treatments usually only relieve itching or reduce irritation caused by external factors, do not cure. Such as glucocorticoids were used to treat inflammatory skin pruritus^19^. In addition, patients need to prolong prescribing, which may lead to drug resistance and adverse side effects. Due to its complex mechanism, pruritus is rarely studied. In this study, in order to explore the therapeutic mechanism of WLZYW in pruritus, network pharmacology was used to find the key agents and target pathways by constructing ingredients-targets-pathway and other networks.

Now the mechanism of itching is thought that degranulation via Lyn in mast cells or basophils results in the phosphorylation and recruitment of Syk, which in turn activates the substrate LAT and NLAT signaling molecules, such as PI3K/AKT, ERK1/2, PLC, PKC, transcription factors, and NF-κB^20^. The activation of these molecules enhances genes expression, the most important is VEGF^21^. Binding of VEGFR to VEGF ligand will directly activate phosphotyrosine residues, such as Sck, PLC-γ, PI3K, Akt, and MAPK^22^. VEGFa is a versatile polypeptide that mediates endothelial cell specific responses, including induced proliferation and vascular leakage. Krause^23^ reported significant improvement in simple pruritus patients treated with bevacizumab (monoclonal VEGF antibody). Moreover, serum levels of VEGF were significantly higher in patients with pruritus^23^, and correlated with severity assessed by physicians. VEGFa can effectively enhance vascular permeability, with molar efficacy approximately 1000-fold greater than that of histamine^24^. In addition, mast cells constitutionally express VEGF as preformed heparin-binding factor^25^ and can secrete this protein to respond to triggers, including IgE and antigen^4^.

After screening drug targets network, 16 core targets were identified following multiple analyses of 160 common targets between WLZYW and pruritus. The results indicated that kaempferol, luteolin, and quercetin might be the key ingredients in WLZYW able to exert therapeutic effects against pruritus. All three are natural flavonoids found in many plants with therapeutic properties, such as antioxidant, anticancer, and anti-inflammatory^26^. Flavonoid derivatives act as antivascular agents by interfering VEGF/VEGFR2 pathway^27^. Kaempferol inhibits VEGF secretion in a time-dependent manner by regulating the signal cascades of PI3K/AKT, MEK and ERK^28,29^. Quercetin can inhibit the secretion of angiogenic factor^30^. Kaempferol and quercetin effectively inhibit the development of IgE-mediated allergic inflammation^31^. Kaempferol prevents MC-mediated allergic diseases by attenuating Lyn activation^32^. ERK1/2 is regulated dose-dependently by luteolin, induced by inhibition of AKT and MAPK pathways^33^. Luteolin can inhibit the FcεRΙ- and MrGPrx2-mediated allergic reaction in vivo and in vitro, and reduce the serum levels of histamine, TNF-α, MCP-1, IL-8 and IL-13 in mice^34^.

GO and KEGG pathways enrichment results indicated that WLZYW might exert effects on pruritus by regulating PI3K-Akt (has04151) signaling pathway. Pathview revealed that WLZYW act not only on AKT, but also on the upstream and downstream of AKT pathway, as well as on VEGF and ERK1/2. Recently, in vivo experiments have confirmed the effects of p-AKT and p-ERK in chronic pruritus, enhance the expression of melatonin and improve the quality of sleep^35^. LY294002 (Akt phosphorylation antagonist) can effectively treat atopic dermatitis in mice and significantly inhibit scratching behaviors^36^. These results suggested that Akt can be targeted in the treatment of pruritus. RBL-2H3 cell line is a useful in vitro model for studying the degranulation of mast cells induced by IgE–FcεRI^37^. ERK1/2 regulates the phosphorylation of cPLA2, as well as the production and release of arachidonic acid, which are the major factors of pruritus^38^. Through in vitro experiments, both concentrations of WLZYW significantly inhibited PI3K/AKT and ERK1/2 signaling pathways, and the degree of degranulation in the model reduced. To understand the relationship between two pathways and VEGFa, wortmannin (1 µM) and U0126 (2 µM) were added to inhibit the model. Western blot and PCR experiments proved WLZYW induced down-regulation of TNF-α and VEGFa mRNA and protein levels. In conclusion, WLZYW downregulates VEGFa expression by inhibiting the phosphorylation of PI3K/AKT and ERK1/2 pathways.

Our results suggest that the active components of WLZYW regulate mast cell activation in many ways, thereby inhibiting the release of sensitizing mediators caused by degranulation, which is main mechanism in the treatment of pruritus. The limitation of this study is that in vivo experimental verification was not carried out. In addition, the etiology of pruritus is complex, and it is difficult to simulate perfectly with only one model.

## Conclusion

Network pharmacology can demonstrate the pharmacological effects of WLZYW on pruritus via multiple components, multiple targets and multiple pathways. The molecular docking results suggest that the main pathway is PI3K/AKT, the main target is VEGFa. In vitro, WLZYW inhibits DNP-IgE/BSA-induced RBL-2H3 degranulation and regulates VEGFa expression by inhibiting PI3K/AKT and ERK1/2 pathways.

## Supplementary Information

Supplementary Legends.

Supplementary Figure 1.

Supplementary Figure 2.

Supplementary Figure 3.

Supplementary Figure 4.

Supplementary Legends.

Supplementary Table 1.

Supplementary Table 2.

Supplementary Table 3.

Supplementary Table 4.

Supplementary Table 5.

Supplementary Table 6.

Supplementary Table 7.

## Data Availability

No large datasets were generated or analyzed during this study. The data used to support the findings of this study are included within the supplementary information files. Minimal datasets necessary to interpret and/or replicate data in this paper are available on request to the corresponding author.
