# Supplementary material for: Molecular docking, network pharmacology and experimental verification to explore the mechanism of Wulongzhiyangwan in the treatment of pruritus
Source: Sci Rep. 2023 Jan 7;13:361. doi: 10.1038/s41598-023-27593-5 (PMC9825397; doi:10.1038/s41598-023-27593-5)
Supplement: Supplementary file 1 — Supplementary Legends. [file 41598_2023_27593_MOESM1_ESM.docx]

Supplementary Table 1: Ten drugs in the WLZYW formula corresponded to 59 complex components, and 235 drug targets were selected by setting OB ≥ 30% and DL ≥ 0.18 for these drugs following the drug-like principle.

Supplementary Table 2: A total of 3660 pruritus-related targets of pruritus obtained by TTD, Drugbank, and GeneCard, were collated.

Supplementary Table 3: A hypergeometric test of the protein interaction results was performed, and statistically significant (p < 0.05) results were obtained, suggesting 2955 edges with 160 nodes.

Supplementary Table 4: A network analysis of 160 targets showed an average degree of centrality of 36.9375 for the compounds, and a total of 60 genes with a centrality degree greater than the median value were selected.

Supplementary Table 5: With an average degree of centrality of 41.73333, these 60 genes were screened again, and 26 genes showed a degree of centrality greater than the aforementioned median value.

Supplementary Table 6: Gene Ontology (GO) analyses were performed with the 26 target genes in the PPI core network, resulting in 2489 GO enriched terms.

Supplementary Table 7: Kyoto Encyclopedia of Genes and Genomes (KEGG) analyses were performed with the 26 target genes in the PPI core network, resulting in 163 KEGG pathways.
