# Supplementary figures and images for: Molecular docking, network pharmacology and experimental verification to explore the mechanism of Wulongzhiyangwan in the treatment of pruritus
Source: Sci Rep. 2023 Jan 7;13:361. doi: 10.1038/s41598-023-27593-5 (PMC9825397; doi:10.1038/s41598-023-27593-5)

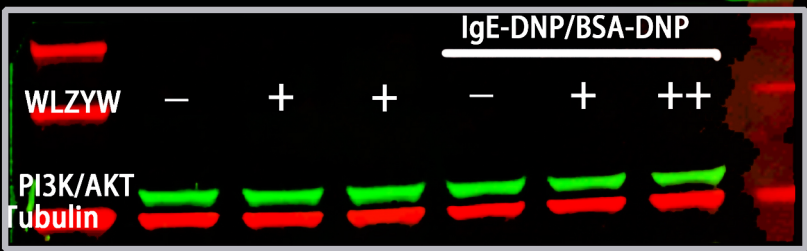

Supplement: Supplementary file 2 — Supplementary Figure 1. [file 41598_2023_27593_MOESM2_ESM.pdf]

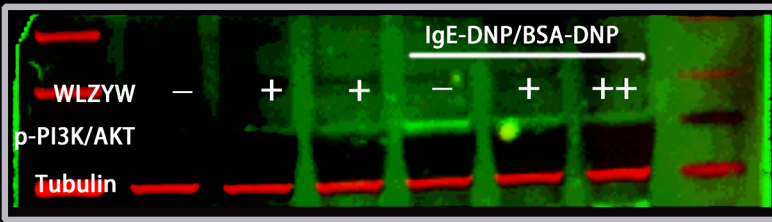

Supplement: Supplementary file 3 — Supplementary Figure 2. [file 41598_2023_27593_MOESM3_ESM.pdf]

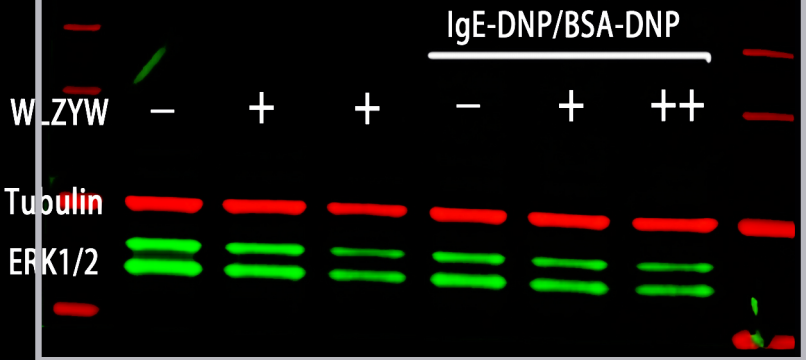

Supplement: Supplementary file 4 — Supplementary Figure 3. [file 41598_2023_27593_MOESM4_ESM.pdf]

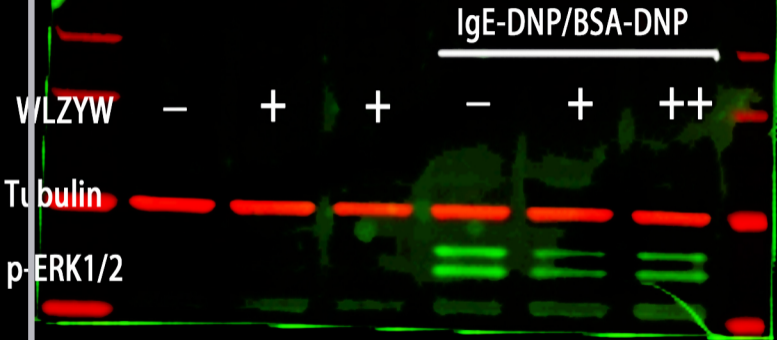

Supplement: Supplementary file 5 — Supplementary Figure 4. [file 41598_2023_27593_MOESM5_ESM.pdf]
