# Supplementary material for: Molecular docking, network pharmacology and experimental verification to explore the mechanism of Wulongzhiyangwan in the treatment of pruritus
Source: Sci Rep. 2023 Jan 7;13:361. doi: 10.1038/s41598-023-27593-5 (PMC9825397; doi:10.1038/s41598-023-27593-5)
Supplement: Supplementary file 6 — Supplementary Legends. [file 41598_2023_27593_MOESM6_ESM.docx]

**Supplement Raw date legends**

**Supplementary Figure 1—SFigure6A-pAKT-WB-rawdata.**  WLZYW 50ug/ml treatment inhibited the phosphorylation levels of p-PI3K/AKT **(A)** in DNP-IgE/BSA induced degranulation model (Mod). The membranes were probed with the anti-p-PI3K/AKT antibody (CST, USA), and a mouse monoclonal antibody against tubulin (Abclonal CHN) overnight at 4 °C. IRDye® 680RD Donkey anti-Mouse (Red) and IRDye 800CW Donkey anti-Rabbit (Green) served as a secondary antibody. The visualization of protein bands was accomplished by Li-cor Odyssey infrared imaging system (LI-COR USA).

**Supplementary Figure 2—SFigure6A-AKT-WB-rawdata.** WLZYW 50ug/ml treatment inhibited the phosphorylation levels of PI3K/AKT in DNP-IgE/BSA induced degranulation model (Mod). The membranes were probed with the anti-PI3K/AKT antibody (CST, USA), and a mouse monoclonal antibody against tubulin (Abclonal CHN) overnight at 4 °C. IRDye® 680RD Donkey anti-Mouse (Red) and IRDye 800CW Donkey anti-Rabbit (Green) served as a secondary antibody. The visualization of protein bands was accomplished by Li-cor Odyssey infrared imaging system (LI-COR USA).

**Supplementary Figure 3—SFigure6B-pERK-WB-rawdata.** WLZYW 50ug/ml treatment inhibited the phosphorylation levels of p-ERK1/2**(B)** in DNP-IgE/BSA induced degranulation model (Mod). The membranes were probed with the anti-p-ERK1/2 antibody (CST, USA), and a mouse monoclonal antibody against tubulin (Abclonal CHN) overnight at 4 °C. IRDye® 680RD Donkey anti-Mouse (Red) and IRDye 800CW Donkey anti-Rabbit (Green) served as a secondary antibody. The visualization of protein bands was accomplished by Li-cor Odyssey infrared imaging system (LI-COR USA).

**Supplementary Figure 4—SFigure6B-ERK-WB-rawdata.** WLZYW 50ug/ml treatment inhibited the phosphorylation levels of ERK1/2**(B)** in DNP-IgE/BSA induced degranulation model (Mod). The membranes were probed with the anti-ERK1/2 antibody (CST, USA), and a mouse monoclonal antibody against tubulin (Abclonal CHN) overnight at 4 °C. IRDye® 680RD Donkey anti-Mouse (Red) and IRDye 800CW Donkey anti-Rabbit (Green) served as a secondary antibody. The visualization of protein bands was accomplished by Li-cor Odyssey infrared imaging system (LI-COR USA).
